# Salivary microRNA 155, 146a/b and 203: A pilot study for potentially non-invasive diagnostic biomarkers of periodontitis and diabetes mellitus

**DOI:** 10.1371/journal.pone.0237004

**Published:** 2020-08-05

**Authors:** Natheer H. Al-Rawi, Farah Al-Marzooq, Ahmed S. Al-Nuaimi, Mahmood Y. Hachim, Rifat Hamoudi

**Affiliations:** 1 Department of Oral & Craniofacial Health Sciences, College of Dental Medicine, University of Sharjah, Sharjah, UAE; 2 Department of Medical Microbiology & Immunology, College of Medicine and Health Sciences, UAE University, Al Ain, UAE; 3 Primary Health Care Corporation (PHCC), Doha, Qatar; 4 College of Medicine, Mohammed Bin Rashid University of Medicine and Health Sciences, Dubai, UAE; 5 Sharjah Institute for Medical Research, College of Medicine, University of Sharjah, Sharjah, UAE; 6 Division of Surgery and Interventional Science, University College London, London, United Kingdom; University of the Pacific, UNITED STATES

## Abstract

Dysregulated expression of MicroRNAs (miRNAs) plays substantial role in the initiation and progression of both diabetes and periodontitis. The aim of the present study was to validate four miRNAs in saliva as potential predictive biomarkers of periodontal disease among patients with and without diabetes mellitus (DM). MiRNAs were extracted from the saliva of 24 adult subjects with DM and 29 healthy controls. Each group was subdivided into periodontally healthy or having periodontitis. In silico analysis identified 4 miRNAs (miRNA 155, 146 a/b and 203) as immune modulators. The expression of miRNAs-146a/b, 155, and 203 was tested using quantitative PCR. The expression levels in the study groups were compared to explore the effect of diabetes on periodontal status and vice versa. In our cohort, the four miRNAs expression were higher in patients with periodontitis and/or diabetes. miRNA-155 was the most reliable predictors of periodontitis among non-diabetics with an optimum cut-off value of < 8.97 with accuracy = 82.6%. MiRNA 146a, on the other hand, was the only reliable predictor of periodontitis among subjects with diabetes with optimum cut-off value of ≥11.04 with accuracy = 86.1%. The results of the present study concluded that MiRNA-146a and miRNA155 in saliva provide reliable, non-invasive, diagnostic and prognostic biomarkers that can be used to monitor periodontal health status among diabetic and non-diabetic patients.

## Introduction

Diabetes mellitus represents an established risk factor for periodontitis. The risk of periodontitis is increased by approximately three folds in diabetic patients compared with non-diabetics [1]. The interrelationship between diabetes and periodontal diseases reflects a vicious circle between oxidative stress and inflammation [2]. Periodontitis is a chronic polymicrobial dysbiotic inflammatory disease of the supporting tissues of the tooth which results in decreased tooth support. Worldwide, periodontal disease affects up to 20% of the population. More than 450 species settle in subgingival pocket [3, 4], including five major bacterial complexes and various microbial species [5]. The innate and adaptive immune responses were involved in periodontal tissue against periodontopathic bacteria [6]. The link between periodontal disease and other general health problems, such as diabetes, adverse pregnancy outcome and cardiovascular diseases have been proclaimed recently [7]. The association between periodontitis and diabetes implicates the potential role of periodontal pathogens on adjustment of inflammation through Micro RNAs (miRNA) [8]. Micro RNAs are family of small (19–24 nucleotides) non-coding RNAs that modulate gene expression and have fundamental biologic functions [9]. Around 300 conserved miRNAs are encoded by the mammalian genome [10], a single miRNA can set hundreds of different messenger RNAs, suggesting that a larger proportion of the transcriptome is subjected to miRNA regulation. Accordingly, miRNAs may rearrange up to 30% of all human genes [11]. During the last decade, miRNAs have materialized as stringent immune response regulators, due to their ability to modify the post-transcriptional expression of multiple genes. At present, more than 100 miRNAs that are selectively expressed in cells involved in innate and adaptive immune response have been specified [12]. MiRNAs are present intracellularly and extracellularly. Extracellular miRNAs, are stable in all body fluids. Recent reports have shown that periodontitis can intone the periodontal tissue levels of miRNAs [13, 14] which may also change miRNA profile in saliva [15]. However, the relationship between salivary miRNAs profile, periodontal condition, and diabetes remains ambiguous. Several miRNAs have been suggested as potential indicators for periodontal disease in many studies [15–17]. To date, there are inconclusive data on the validity of salivary miRNAs and the risk of periodontitis among patients with diabetes. Therefore, our aim in the present study was to identify the accuracy of four selected miRNAs in saliva that could accurately predict the risk of periodontitis among patients with diabetes and non-diabetic subjects.

## Material and methods

### Bioinformatics search for candidate salivary miRNA markers in periodontal diseases and diabetes mellitus

**Fig 1:**
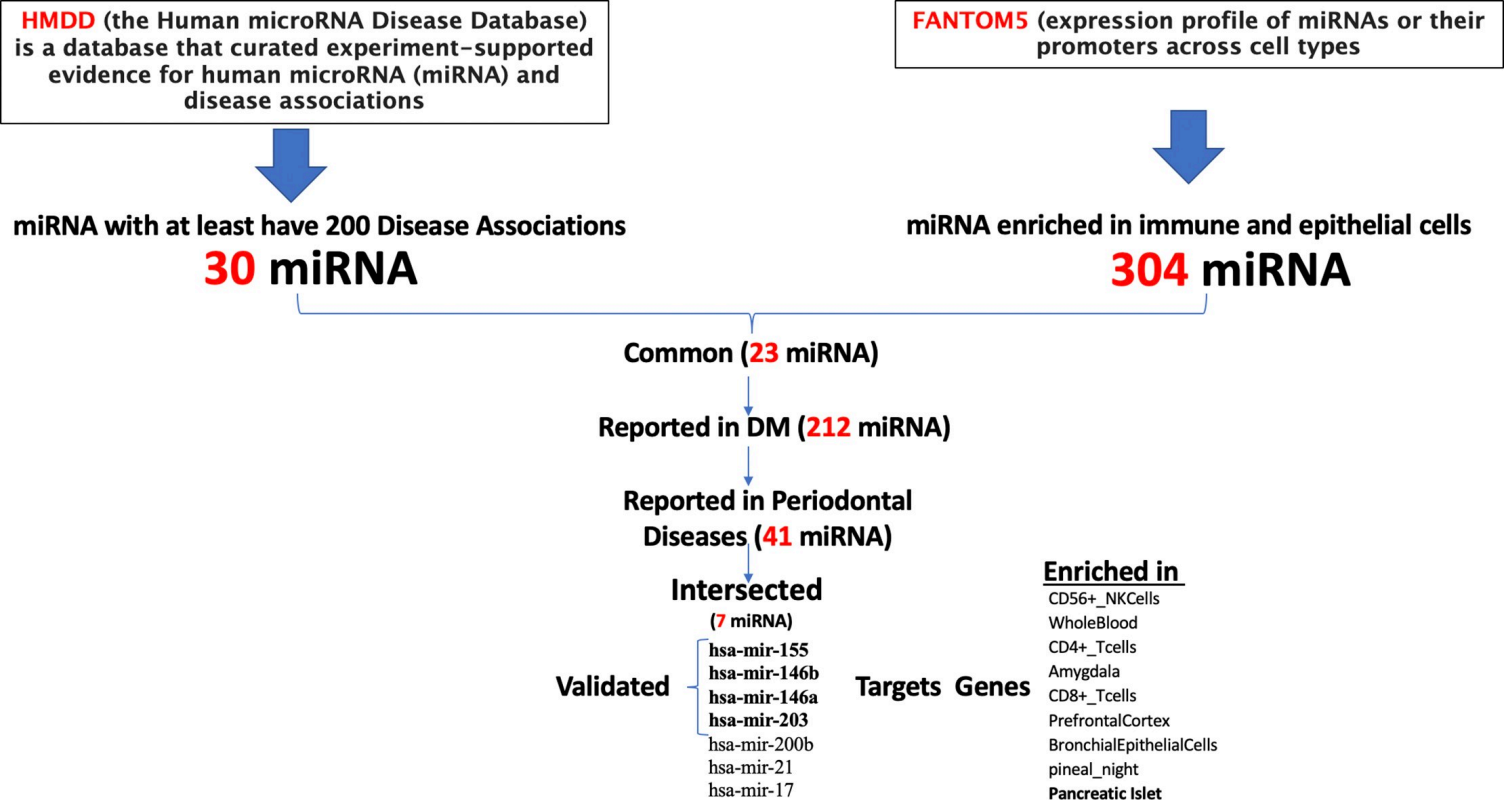
Flowchart for bioinformatics search for candidate salivary miRNA markers in periodontal diseases and diabetes mellitus.

**Fig 2:**
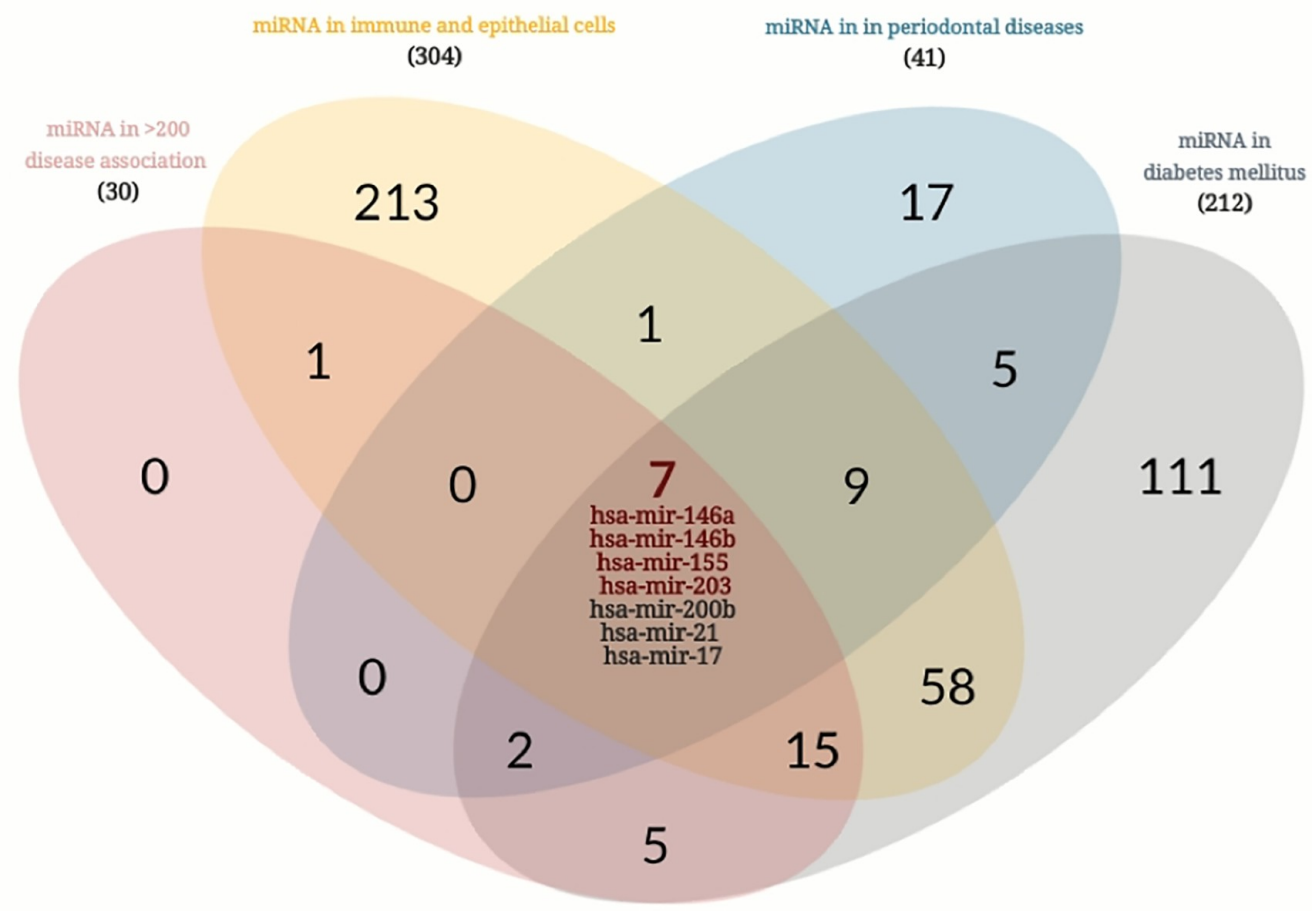
Venn diagram of bioinformatics search for candidate miRNA markers in periodontitis and diabetes mellitus.

In order to identify candidate miRNA that can serve as a potential biomarker in the saliva of patients with periodontal diseases and diabetes mellitus, a comprehensive search was done using publicly available databases. As shown in the flowchart in Fig 1 and Venn diagram in Fig 2, the Human microRNA Disease Database (HMDD) of curated experiment-supported evidence for human microRNA (miRNA) and disease associations was downloaded and searched for the top miRNA that has at least 200 documented diseases association. This database contains a manually collected 35547 miRNA-disease association (1206 miRNA genes, 893 diseases from 19280 papers) [18]. Then we looked for another comprehensive database FANTOM5 (Functional ANnoTation of the Mammalian genome) to explore the expression profile of miRNAs or their promoters across cell types [19]. We looked for miRNAs that are specifically enriched in immune and epithelial cells as they represent most cells in saliva samples of subjects with periodontitis and diabetes mellitus. Then, we intersected the two resultant shortlisted miRNAs, and further filtered them to those that enriched in periodontal diseases and diabetes mellitus only. The resultant miRNA targeted genes were searched for the cell/tissue/organ specificity using Enricher gene list enrichment analysis tool [20]. In order to validate the involvement of the identified miRNAs in the pathogenesis of periodontitis and its progression, we searched if the predicted gene targets of the hsa-mir-146a, hsa-mir-146b, hsa-mir-155, and hsa-mir-203 have any specific pathway enrichment. The predicted gene targets for each miRNA were identified using target scan tool (http://www.targetscan.org/vert_72/). The targeted genes were analyzed for functional pathway analysis using DAVID Bioinformatics Resources 6.8, NIAID/NIH Functional Annotation Tool (https://david.ncifcrf.gov/summary.jsp). S1 File provides a list of all the bioinformatics tools used in this study.

### Study participants

Fifty-three subjects (27 males and 26 females, mean age 36.26 ± 13.57 year), chosen among individuals with diabetes (n = 24) and non-diabetics (n = 29) were included in the study. All procedures performed in this study were fully explained to the participants and the written consents were obtained. The study protocol was approved by the Institutional Review Board of the University of Sharjah under the number (REC-16-10-12-S) and with the 1964 Helsinki declaration and its later amendments.

The participants from University Dental Hospital of Sharjah between November 2017 and March 2018. The participants were recruited from UAE population. Participants with body mass index (BMI) <25, and more than 20 erupted teeth excluding third molars were included in the study.

The consented participants were interviewed for full medical history taking. Routine periodontal examination was done by the registered periodontist (AA). The study participants included 24 controlled diabetic patients (HbA1c <8%) and 29 non-diabetic patients as shown in Table 1.

**Table 1:** Demographic data of the studied sample.

Study groups	Age (years)	Participants (Males/females)	BMI (Kg/cm^2^)	HBA1C
Healthy control	25.92 ± 6.72	7/7 (14)	22.84 ± 0.89	6.30 ± 1.25
Periodontitis only	40.46 ± 12.46	7/8 (15)	23.86 ± 1.01	6.45 ± 1.46
Diabetes with healthy periodontium	32.4 ± 14.38	6/4 (10)	24.05 ± 1.25	7.01 ± 0.97
Diabetes with periodontitis	49.58 ± 6.34	7/7 (14)	24.2 ± 1.06	7.14 ± 0.85

The index teeth in each sextant were examined by running the WHO community periodontal index probe around the entire sulcus of each tooth and the highest score recorded. Subjects with healthy periodontium should have gingival index = 0 and probing depth less than 3 mm. in every site [21]. Subjects with chronic periodontitis should have pocket depth equal or more than 5 mm at two or more sites with clinical attachment loss of more than 4 mm in two or more interproximal sites (not on the same tooth) [22]. Subjects with history of any systemic acute or chronic inflammatory diseases, smoking, on chronic medications or on dietary supplementation were excluded from the study.

### Saliva collection

Patients were asked to refrain from drinking eating, and tooth brushing one hour prior to sample collection. To remove any food residue, mouth rinsing with tab water was first done, then the sample was collected. Using passive drool method, 2–5 ml of unstimulated whole saliva was collected from each patient. The collected saliva was centrifuged (2500g for 10 minutes) at room temperature. Supernatant was isolated and stored at -80ºC until further analyzed.

#### RNA extraction

RNA was extracted from the saliva samples using a commercial kit (Total RNA Purification Plus Kit; Norgen, Canada)RNA was reverse transcribed using MystiCq^®^ microRNA cDNA Synthesis Mix (Sigma, USA). For miRNA expression assays, the following forward primers were used (Sigma, USA) for the quantification of miRNA146a (hsa-miR-146a-5p), miRNA146b (hsa-miR-146b-3p), miRNA155 (hsa-miR-155-3p), and miRNA203 (hsa-miR-203). A universal reverse primer provided with the MystiCq cDNA reverse transcription kit was used for all miRNA expression analysis. The qPCR signal for miRNA was normalized by comparison with a small nuclear RNA (snRNA) U6 as a housekeeping gene. Real-time PCR was carried out with a StepOne Real-Time PCR thermocycler (Applied biosystem, USA). Amplification was performed in a total reaction volume of 20 μl containing 5x HOT FIREPol^®^ EvaGreen^®^ qPCR Mix Plus (Solis BioDyne, Estonia). Each sample was tested in duplicate.

#### Real-time PCR analysis

The cycle threshold value “ΔCt” for each sample was determined by subtracting the Ct value of the target miRNA gene from that of the housekeeping gene of the same sample (small nuclear RNA (snRNA) U6 for miRNA quantification), i.e; ΔCt  =  Ct target DNA − Ct housekeeping gene. For the comparison between different study groups, ΔΔCt was calculated:

ΔΔCt = ΔCt value of the sample for a particular target—average ΔCt of the control group for the same target.

Relative expression of miRNA was calculated using the 2^-ΔΔCt^ method [23]. The comparator Ct method of four micro RNAs was used to assess three effects in the present study. These include a comparison between diabetic subjects and non-diabetic subjects with healthy and diseased periodontium; comparison between diabetic subjects and non-diabetic subjects with periodontitis, and comparison between diabetic subjects with healthy periodontium with those with periodontitis. The standardized Ct value for a specific marker was tested for its ability to predict a specific outcome (diagnosis) differentiating it from its absence (control group). Therefore, a lower standardized Ct value indicates that a higher expression of the marker is predictive of the outcome, while a higher standardized Ct value indicates that a lower expression of the marker is predictive of the outcome. To remove the experimental artifacts, the raw Ct values of housekeeping gene (namely U6) was subtracted from the raw Ct value of the selected miRNAs. The difference in mean standardized Ct value of a specific miRNA between a target group and a comparison (reference) group was used as a negative power for a multiplier of two-fold to show the fold change imposed by a specific effect.

#### Statistical analysis

Area under the curve (AUC) using receiver operating characteristic (ROC) was calculated for miRNA 146 a & b, miRNA 155, and miRNA 203. A 2-tailed p-value <0.05 was considered statistically significant. All statistical analyses were performed using SPSS for Windows software version 23.0 (SPSS Inc, IL, USA).

The validity parameters for selected cutoff values of the tested parameters were calculated using ROC method. The optimal cutoff value for the tested parameters corresponds to the shortest distance on the ROC curve, estimated at each one-half unit of the tested index. A distance on the ROC curve is equal to $\sqrt{{\left(1-sensitivity\right)}^{2}+{\left(1-specificity\right)}^{2}}$ [24].

## Results

This case-control study consisted of 53 adult subjects, (27 males and 26 females). The periodontal status of the studied subjects was assessed by community Periodontal index (CPI). We found that 14 (58.3%) of diabetic subjects and 15 (51.7%) of non-diabetic subjects (control) were suffering from mild to moderate periodontitis (loss of attachment 1–3 mm in more than one site).

### hsa-mir-146a, hsa-mir-146b, hsa-mir-155, and hsa-mir-203 are potential biomarkers in periodontal diseases and diabetes

Using the HMDD database, our bioinformatics search was done which identified the top 35 miRNAs that are associated with at least 200 different diseases. FANTOM5 databases were also used, which identified 304 miRNAs that were significantly enriched in immune and epithelial cells. 23 top miRNAs were shown to be enriched in immune and epithelial cells and further filtered into 7 miRNAs that showed enrichment in periodontal diseases and diabetes, the top four were selected in this study which were hsa-mir-146a, hsa-mir-146b, hsa-mir-155, and hsa-mir-203. Interestingly, the gene targets of those miRNA were shown to be enriched in CD56+ NK cells, whole blood, CD4+ T cells, CD8+ T cells, and pancreatic islet as shown in Figs 1 and 2.

**Fig 3:**
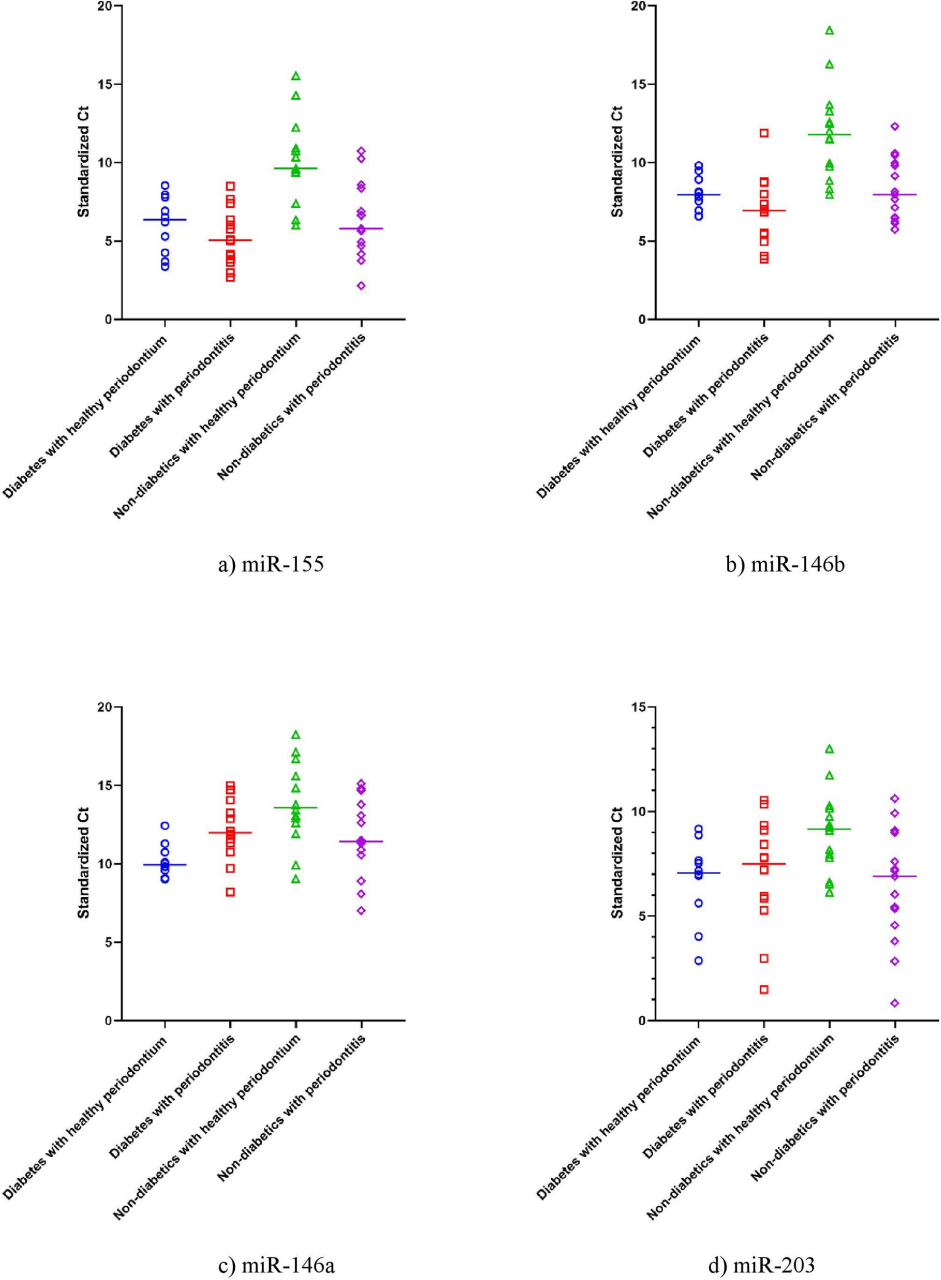
The expression of miRNA a) miR-155, b) miR-146b, c) miR-146a and d) miR-203 in the study groups. Data shown is the standardized Ct for each miR in each participant of the study (n = 53). The horizontal line represents the mean Ct value for each group. Ct is inversely proportional to the gene expression; thus, the lower the CT value, the higher the expression level.

Using the housekeeping gene (U6) as a normalization control, we standardized the Ct values obtained from each subject. Fig 3 summarized the expression of miRNA 155, 146a and b, and miRNA 203 based on the standardized Ct values of each miRNA. The lower the Ct value the higher the expression of the indicated miRNA in the saliva of the subject.

#### MiRNA 155 expression

We found that the miRNA-155 expression was 16.5 folds higher among diabetics compared to non-diabetic subjects with healthy periodontium. This reflects the change of MiRNA 155 expression due to diabetes. Similarly, non-diabetic subjects with periodontitis has significantly higher expression of miRNA-155 by 13.7 folds compared to similar subjects with healthy periodontium. This revealed the change of miRNA 155 expression due to periodontitis among non-diabetic subjects. Interestingly, the marginal increase in miRNA-155 expression attributed to periodontitis was not statistically significant among diabetic group. MiRNA 155 expression was 29.3 folds higher among diabetic subjects with unhealthy periodontium when compared with non-diabetic subjects with healthy periodontium. This reflects the combined changes of miRNA155 expression due to both diabetes and periodontitis. To measure the changes in miRNA 155 expression that is due to diabetes in patients with periodontitis, we did a comparison between subjects with diabetes and unhealthy periodontium and healthy subjects with periodontitis only which revealed 2.1 fold increase in miRNA 155 expression, however this increase in miRNA 155 expression was not statistically significant.(p>0.05), as shown in Table 2.

**Table 2:** The difference between the four study groups in standardized Ct value (ΔCt) for miRNA-155, miRNA 146b, miRNA 146a and miRNA 203.

Study groups		Mir-155				Mir-146b				Mir-146a				Mir-203			
		Range	Mean	SD	SE	Range	Mean	SD	SE	Range	Mean	SD	SE	Range	Mean	SD	SE
**Diabetes**	healthy periodontium (n = 10)	3.38–8.54	6.05	1.84	0.58	6.58–9.81	7.99	1.14	0.36	9.02–12.43	10.19	1.04	0.33	2.87–9.17	6.68	1.99	0.63
	periodontitis (n = 14)	2.68–8.49	5.23	1.8	0.48	3.85–11.88	6.9	2.12	0.57	8.19–14.98	12.28	1.98	0.53	1.49–10.54	7.09	2.62	0.7
**Non-diabetics**	healthy periodontium (n = 14)	6–15.54	10.1	2.68	0.72	7.96–18.45	11.91	2.97	0.79	9.04–18.24	13.78	2.59	0.69	6.14–13.01	8.99	1.97	0.53
	Periodontitis (n = 15)	2.15–10.73	6.32	2.38	0.61	5.74–12.31	8.29	2	0.52	7.02–15.11	11.67	2.43	0.63	0.84–10.62	6.43	2.71	0.7
P (ANOVA)		<0.001				<0.001				0.002				0.029			
**Effect of diabetes (Diabetes with healthy periodontium vs. non-diabetic with healthy periodontium**																	
ΔΔCt		-4.05				-3.92				-3.58				-2.3			
Fold change (2^-ΔΔCt^)		16.5 ↑				15.1 ↑				12 ↑				4.9 ↑			
P value		<0.001				<0.001				<0.001				0.024			
**Effect of periodontitis among diabetics (Diabetic with unhealthy periodontium vs. diabetic with healthy periodontium)**																	
ΔΔCt		-0.83				-1.09				2.09				0.41			
Fold change (2^-ΔΔCt^)		1.8 ↑				2.1 ↑				0.24 ↓				0.75 ↓			
P value		0.38 [NS]				0.24 [NS]				0.024				0.68 [NS]			
**Effect of periodontitis among non-diabetics (Non-diabetic with unhealthy periodontium vs. non-diabetic with healthy periodontium)**																	
ΔΔCt		-3.77				-3.62				-2.1				-2.56			
Fold change (2^-ΔΔCt^)		13.7 ↑				12.3 ↑				4.3 ↑				5.9 ↑			
P value		<0.001				<0.001				0.012				0.006			
**Effect of diabetes combined with periodontitis (diabetic/ unhealthy periodontium x non-diabetic/ healthy periodontium)**																	
Delta Delta Ct		-4.87				-5.01				-1.50				-1.89			
Fold change (2^-delta delta Ct)		29.3↑				32.2↑				2.8↑				3.7↑			
P value		<0.001				<0.001				0.07[NS]				0.041			
				
**effect of diabetes on patients with periodontitis (diabetic/ unhealthy periodontium x non diabetic/ unhealthy periodontium) **																	
Delta Delta Ct		-1.10				-1.39				0.60				0.67			
Fold change (2^-delta delta Ct)		2.1↑				2.6↑				0.7↓				0.6↓			
P value		0.19[NS]				0.1[NS]				0.46[NS]				0.46[NS]			

[NS]: non-significant difference.

↑ over-expressed, ↓ under-expressed.

Ct: Cycle threshold.

SD: Standard Deviation.

SE: Standard error.

#### MiRNA 146b expression

MiRNA 146 b expression was 15.1 folds higher among diabetic subjects with healthy periodontium when compared with non-diabetic subjects with healthy periodontium. Similarly, MiRNA 146b expression was higher by a mean of 12.3 folds when non-diabetic subjects with periodontitis is compared with non-diabetic subjects with healthy periodontium. Regarding the effect of periodontitis among diabetics, a non-significant statistical difference in miRNA146b expression was found between diabetic subjects with periodontitis and diabetic subjects with healthy periodontium. MiRNA 146b expression was 32.2 folds higher among diabetic subjects with unhealthy periodontium when compared with non-diabetic subjects with healthy periodontium and was 2.6 folds higher among subjects with diabetes with periodontitis when compared with healthy subjects with periodontitis, however this increase in miRNA 146a was not statistically significant. (p>0.05), as shown in Table 2.

#### MiRNA 146a expression

MiRNA 146 an expression, on the other hand, was also significantly higher by 12.0 folds among diabetic subjects with healthy periodontium when compared with non-diabetic subjects with healthy periodontium. Similarly, miRNA 146a expression was significantly higher by a mean of 4.3 times when non-diabetic subjects with periodontitis are compared with non-diabetic subjects with healthy periodontium. Regarding the effect of periodontitis among diabetics, a significant statistical difference in miRNA146a under expression was found between diabetic subjects with periodontitis and diabetic subjects with healthy periodontium. MiRNA 146a expression was 2.8 folds higher among diabetic subjects with unhealthy periodontium when compared with non-diabetic subjects with healthy periodontium and was 0.7 folds lower among subjects with diabetes with periodontitis when compared with healthy subjects with periodontitis, however this increase in miRNA 146a was not statistically significant. (p>0.05), as shown in Table 2.

#### MiRNA 203 expression

The expression of miRNA 203 was elevated by a mean of 4.9 folds among diabetic and non-diabetic subjects with healthy periodontium, and an elevation by a mean of 5.9 folds among non-diabetic subjects with periodontitis compared with those without periodontitis. Similar to miRNA 155 and 146b, periodontal health status has a non-significant effect on the expression of miRNA 203 when diabetic subjects with periodontitis were compared with those with healthy periodontium. MiRNA 203 expression was 3.7 folds higher among diabetic subjects with unhealthy periodontium when compared with non-diabetic subjects with healthy periodontium (p<0.04) and was 0.6 folds lower among subjects with diabetes with periodontitis when compared with healthy subjects with periodontitis, however this increase in miRNA 146a was not statistically significant. (p > 0.05), as shown in Table 2.

### Discriminant analysis

The ROC method was also used to rank the tested markers in their ability to discriminate between diabetic and non-diabetic subjects with healthy periodontium. The Larger area under ROC curve, the stronger the miRNA being affected by diabetes.

#### A. Validity parameters for selected markers when used as a test to predict a diagnosis of diabetes among subjects with healthy periodontium

**Fig 4:**
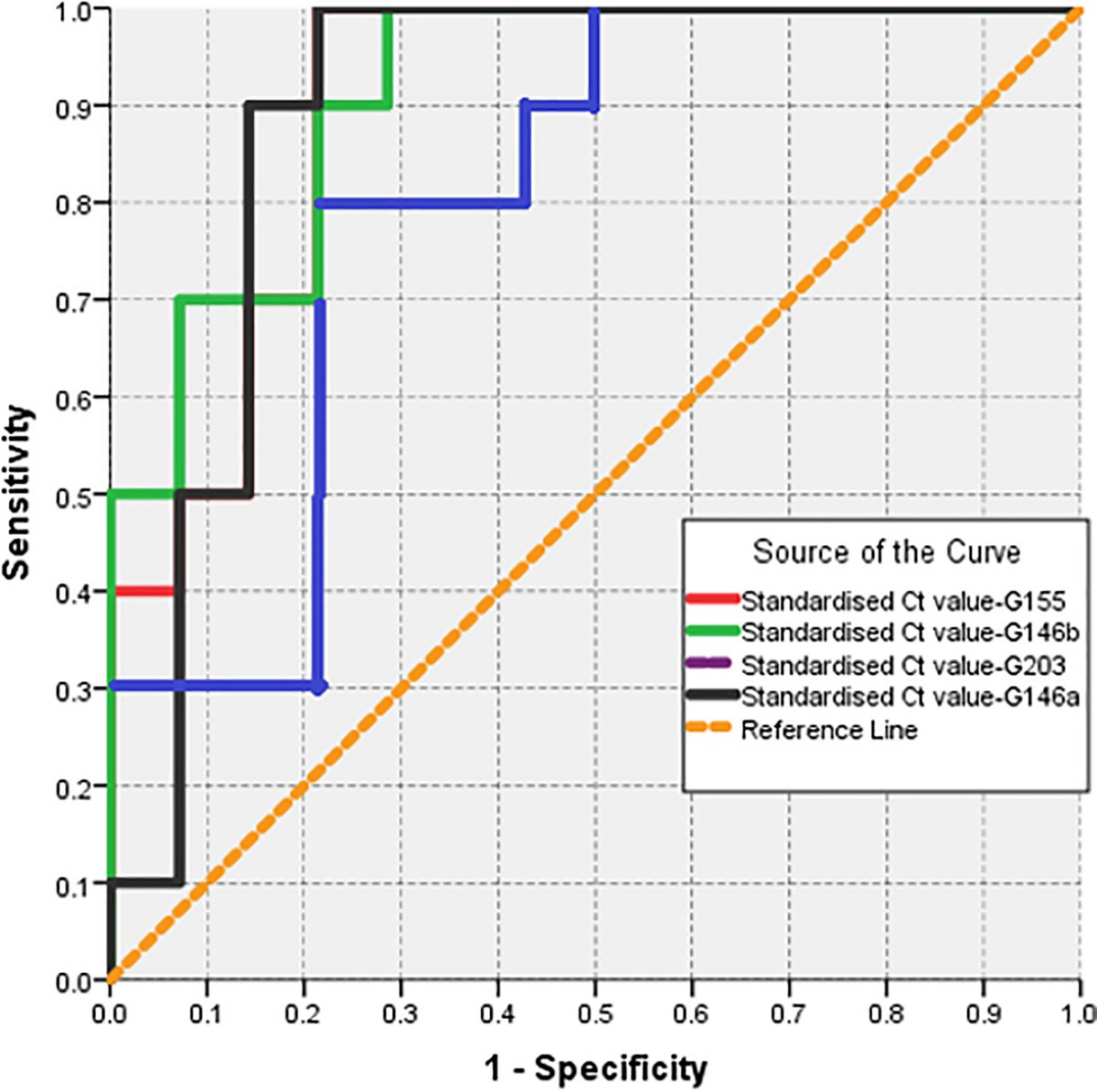
ROC curve for selected markers when used as test to predict a diagnosis of DM among subjects with healthy gum (smaller values of the marker are predictive of a positive diagnosis).

As shown in Table 3 and Fig 4, three miRNAs, namely miR146b, miR155 and miR146a ranked first with the largest area under ROC curve (>0.9). These represent the markers being highly affected by diabetes among those with healthy periodontium. The miR 203 ranked fourth in magnitude of being influenced by diabetes with statistically significant ROC area of >0.8. Accordingly, the optimum cut-off value used to predict a diagnosis of diabetes among subjects with healthy periodontium is calculated. The optimum cut-off value would be a standardized Ct value <8.95 (accuracy = 89.3%) for miR155. For miR 146a the optimum cut-off value was a standardized Ct value <11.32 (accuracy = 86.6%). For miR146b the optimum cut-off value was a standardized Ct value <9.34 (accuracy = 83.0%) and for miR 203 the optimum cut-off value was a standardized Ct value <7.66 (accuracy = 76.8%) as shown in Table 4.

**Table 3:** Area under ROC curve (AUC) for selected markers when used as a test to predict a diagnosis of diabetes among subjects with healthy periodontium, predict periodontitis among subjects with diabetes, predict periodontitis among subjects with no diabetes, or predict diabetes among subjects with periodontitis.

Condition predicted by the selected biomarker	Biomarkers	mir146b	mir155	mir146a	mir 203
**diabetes among subjects with healthy periodontium**	AUC	0.92	0.902	0.902	0.804
	P value	0.001	0.002	0.002	0.02
**Periodontitis among subjects with diabetes**	AUC	0.70	0.65	0.82	0.62
	P value	0.1 (NS)	0.22 (NS)	0.01	0.35(NS)
**periodontitis among subjects with no diabetes**	AUC	0.85	0.862	0.719	0.779
	P value	0.001	<0.001	0.045	0.011
**diabetes among subjects with periodontitis**	AUC	0.681	0.638	0.595	0.590
	P value	0.1 (NS)	0.21 (NS)	0.38 (NS)	0.41(NS)

**Table 4:** Validity parameters for selected markers when used as a test to predict a diagnosis of diabetes among subjects with healthy periodontium (smaller values of the marker are predictive of a positive diagnosis).

Positive if < cut-off value	Sensitivity	Specificity	Accuracy	PPV at pretest probability =		NPV at pretest probability = 10%
				50%	90%	
**Standardized Ct value mir155**						
5.645 (Highest specificity)	50.0	100.0	75.0	100.0	100.0	94.7
8.950 *(Optimum cut-off value) and highest sensitivity)*	100.0	78.6	89.3	82.4	97.7	100.0
**Standardized Ct value mir146b**						
7.895 (Highest specificity)	50.0	100.0	75.0	100.0	100.0	94.7
9.340 *(Optimum cut-off value)*	87.5	78.6	83.0	80.3	97.4	98.3
9.885 (Highest sensitivity)	100.0	71.4	85.7	77.8	96.9	100.0
**Standardized Ct value mir 203**						
5.880 (Highest specificity)	37.5	100.0	68.8	100.0	100.0	93.5
7.665 *(Optimum cut-off value)*	75.0	78.6	76.8	77.8	96.9	96.6
9.205 (Highest sensitivity)	100.0	50.0	75.0	66.7	94.7	100.0
**Standardized Ct value mir146a**						
9.030 (Highest specificity)	12.5	100.0	56.3	100.0	100.0	91.1
11.320 *(Optimum cut-off value)*	87.5	85.7	86.6	86.0	98.2	98.4
12.520 (Highest sensitivity)	100.0	78.6	89.3	82.4	97.7	100.0

#### B. Validity parameters for selected markers when used as test to predict periodontitis among subjects with diabetes

When ROC method was used to predict periodontitis among subjects with diabetes, only miRNA146a was found to have a magnitude of being influenced by periodontitis with statistically significant ROC area of >0.8. As shown in Table 5, the optimum cut-off value of miRNA 146a that predict periodontitis among subjects with diabetes is equal or more than 11.04 (accuracy = 86.1%).

**Table 5:** Validity parameters for selected markers when used as test to predict periodontitis among subjects with diabetes.

	Sensitivity	Specificity	Accuracy	PPV at pretest probability =		NPV at pretest probability = 10%
				50%	90%	
**Standardized Ct value mir155 (Positive if < cut-off value)**						
3.175 (Highest specificity)	14.3	100.0	57.1	100.0	100.0	91.3
6.120 *(Optimum cut-off value)*	71.4	60.0	65.7	64.1	94.1	95.0
8.515 (Highest sensitivity)	100.0	10.0	55.0	52.6	90.9	100.0
**Standardized Ct value mir146b (Positive if < cut-off value)**						
6.040 (Highest specificity)	35.7	100.0	67.9	100.0	100.0	93.3
7.445 *(Optimum cut-off value)*	71.4	70.0	70.7	70.4	95.5	95.7
8.840 (Highest sensitivity)	92.9	30.0	61.4	57.0	92.3	97.4
**Standardized Ct value mir 203 (Positive if ≥ cut-off value)**						
2.902 (highest specificity)	92.3	12.5	52.4	51.3	90.5	93.6
7.100 *(Optimum cut-off value)*	69.2	62.5	65.9	64.9	94.3	94.8
9.255 (Highest specificity)	23.1	100.0	61.5	100.0	100.0	92.1
**Standardized Ct value-mir146a (Positive if ≥ cut-off value)**						
9.335 (Highest sensitivity)	92.3	25.0	58.7	55.2	91.7	96.7
11.040 *(Optimum cut-off value)*	84.6	87.5	86.1	87.1	98.4	98.1
12.655 (Highest specificity)	46.2	100.0	73.1	100.0	100.0	94.4

#### C. Validity parameters for selected markers when used as a test to predict periodontitis among subjects with no diabetes

**Fig 5:**
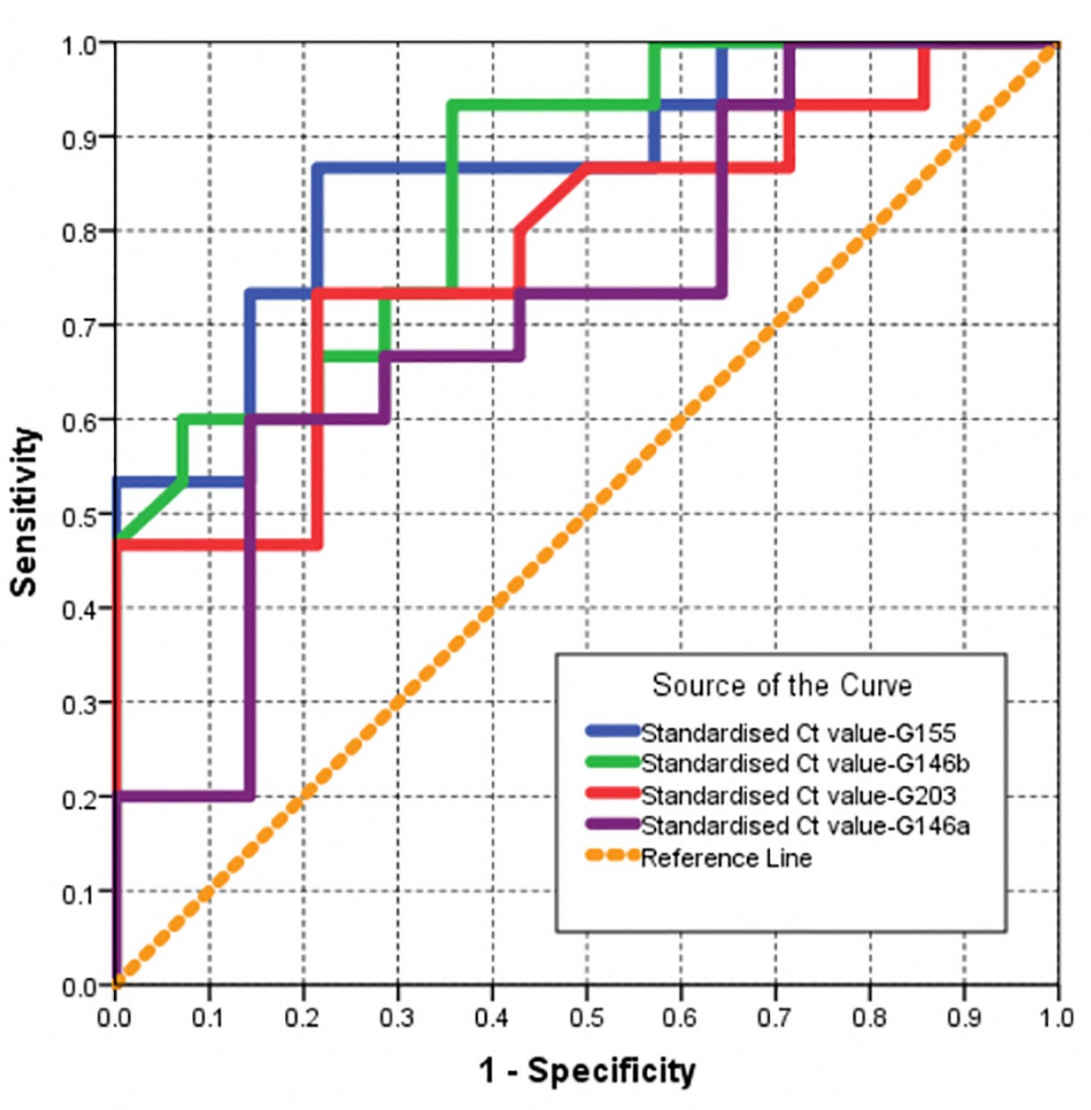
ROC curve for selected markers when used as test to predict periodontitis among subjects with no DM (smaller values of the marker are predictive of a positive diagnosis).

MiR155 and miR 146b were associated with a larger area under ROC curve (>0.8). Those represent markers that predict periodontitis among non-diabetic subjects with optimum cutoff value of 8.97 (accuracy = 82.6%) and 11.04 (accuracy = 78.8%) or less respectively as shown in Table 6 and Fig 5.

**Table 6:** Validity parameters for selected markers when used as a test to predict periodontitis among subjects with no diabetes (smaller values of the marker are predictive of a positive diagnosis).

Positive if < cut-off value	Sensitivity	Specificity	Accuracy	PPV at pretest probability =		NPV at pretest probability = 10%
				50%	90%	
**Standardized Ct value-mir155**						
5.890 (Highest specificity)	53.3	100.0	76.7	100.0	100.0	95.1
8.975 *(Optimum cut-off value)*	86.7	78.6	82.6	80.2	97.3	98.1
10.740 (Highest sensitivity)	100.0	35.7	67.9	60.9	93.3	100.0
**Standardized Ct value-mir146b**						
7.810 (Highest specificity)	46.7	100.0	73.3	100.0	100.0	94.4
11.040 *(Optimum cut-off value)*	93.3	64.3	78.8	72.3	95.9	98.9
12.400 (Highest sensitivity)	100.0	42.9	71.4	63.6	94.0	100.0
**Standardized Ct value-mir203**						
6.090 (Highest specificity)	46.7	100.0	73.3	100.0	100.0	94.4
7.705 *(Optimum cut-off value)*	73.3	78.6	76.0	77.4	96.9	96.4
11.175 (Highest sensitivity)	100.0	14.3	57.1	53.8	91.3	100.0
**Standardized Ct value-mir146a**						
8.970 (Highest specificity)	20.0	100.0	60.0	100.0	100.0	91.8
11.705 *(Optimum cut-off value)*	60.0	85.7	72.9	80.8	97.4	95.1
15.350 (Highest sensitivity)	100.0	28.6	64.3	58.3	92.6	100.0

#### D. Validity parameters for selected markers when used as a test to predict a diagnosis of diabetes among subjects with periodontitis (smaller values of the marker are predictive of a positive diagnosis)

None of the miRNAs were successful to predict the effect a diagnosis of diabetes among subjects with periodontitis. The largest area under curve was for MiR 146b (0.681) which yield in optimum cut off value of (7.50) with low sensitivity (71.4%), specificity (60%) and accuracy (65.7%), as seen in Table 7.

**Table 7:** Validity parameters for selected markers when used as a test to predict a diagnosis of diabetes among subjects with periodontitis.

**Positive if < cut-off value**	**Sensitivity**	**Specificity**	**Accuracy**	**PPV at pretest probability =**		**NPV at pretest probability = 10%**
				**50%**	**90%**	
**Standardized Ct value-mir155**						
3.695 (Highest specificity)	21.4	93.3	57.4	76.3	96.7	91.4
**5.360 (optimum cut off)**	57.1	66.7	61.9	63.2	93.9	93.3
10.490 (Highest sensitivity)	100.0	6.7	53.3	51.7	90.6	100.0
**Standardized Ct value-mir146b**						
3.950 (Highest specificity)	7.1	100.0	53.6	100.0	100.0	90.6
**7.505 (optimum cut off)**	71.4	60.0	65.7	64.1	94.1	95.0
12.095 (Highest sensitivity)	100.0	6.7	53.3	51.7	90.6	100.0
**Positive if ≥ cut-off value**	**Sensitivity**	**Specificity**	**Accuracy**	**PPV at pretest probability =**		**NPV at pretest probability = 10%**
				**50%**	**90%**	
**Standardized Ct value-mir203**						
1.165 (Highest sensitivity)	100.0	6.7	53.3	51.7	90.6	100.0
**7.185 (optimum cut off)**	64.3	60.0	62.1	61.6	93.5	93.8
10.135 (Highest specificity)	14.3	93.3	53.8	68.2	95.1	90.7
**Standardized Ct value-mir146a**						
8.135 (Highest sensitivity)	100.0	13.3	56.7	53.6	91.2	100.0
**11.540 (optimum cut off)**	71.4	60.0	65.7	64.1	94.1	95.0
14.885 (Highest specificity)	7.1	93.3	50.2	51.7	90.6	90.0

## Discussion

Saliva has become very popular as a diagnostic biofluid and a source of biomarkers in many oral as well as systemic disease. Using miRNAs as a non-invasive diagnostic and progression markers for periodontitis could be a promising approach [25]. A wide set of biological functions have been regulated by miRNAs, such as developmental timing, cell differentiation and cancer development [26]. Recent studies suggested that microbial infections can modulate miRNAs expression [27]. The crucial roles of miRNAs found in saliva point out their possible use as biomarkers for diseases promote the discovery of miRNA-based therapeutics [11].

Our bioinformatics search identified the four miRNAs (miRNA 146a and b, 155, and 203) as promising biomarkers for periodontal diseases and diabetes (Figs 2 and 3). Interestingly, the gene targets of these miRNAs are enriched in CD56+ NK cells, whole blood, CD4+ T cells, CD8+ T cells, and pancreatic islet, indicating its potential role in diabetes and immune-related inflammation like periodontal diseases. The gene targets of the four miRNAs are enriched in immune-related pathways. Interestingly, the top significantly enriched pathways were related to immune response triggered by bacterial lipopolysaccharide, positive regulation of T cell proliferation, monocyte chemotaxis, interleukin-1-mediated signaling pathway, positive regulation of interleukin-8 production and negative regulation of chemokine-mediated signaling pathway [27]. Therefore, these four immunomodulatory miRNAs were investigated in patients’ saliva by quantitative real-time PCR to test their validity as indicators of periodontal inflammation among patients with or without type 2 diabetes, or as predictors of diabetes in patients with or without periodontal disease.

The examined miRNAs were overexpressed particularly in the patients suffering from periodontitis, especially if the patient had diabetes. The maximum effect was noted in miRNA 146b and 155. Additionally, the effect of diabetes on the expression of various miRNA was more evident in patients lacking periodontitis. Both diabetes and periodontitis are factors affecting the expression of miRNA, and their effect can be cumulative as seen in patients suffering from both diabetes and periodontitis with the highest expression levels of miRNA 146b and 155. Duval et al suggested that microbial infections induce the expression of specific sets of miRNAs, that are upregulated in a time-dependent manner [27].

MiRNA-155 and miRNA-146 are induced by the nuclear factor kappa B (NF-κB) pathway through pattern recognition receptors (PRR) sensing of pathogen motifs, in particular, LPS [28]. MiRNA146-a is known to play an important role in innate immunity via regulation of cytokine production in response to bacterial endotoxins production [29, 30]. The upregulated miRNA 146a may boost the initial LPS threshold required for miRNA 155 activation [28]. MiRNA 155 limits the production of inflammatory cytokines through regulating TLR-mediated NF-kB activation [31, 32]. During inflammatory responses, the NF-kB-miRNA 155 axis coordinates with NF-kB-miRNA-146a axis to regulate the intensity and duration of inflammation. The combinatorial action of positive (NF-kB-miRNA-155) and negative (NF-kB-miR-146a) regulatory loops provides optimal NF-kB activity during inflammatory stimuli and eventually leads to the resolution of the inflammation. Thus, miRNA-155 and miRNA-146 can cross-talk and regulate inflammatory responses [33].

These facts may explain our observation of high expression levels of miRNA 155 and miRNA 146 (a and b) in patients with periodontitis, due to the progressive inflammation induced by multiple microbes involved in the infection.

Radovic et al investigated the levels of miRNA 146a and miRNA155 in gingival crevicular fluid (GCF) of diabetic and non-diabetic patients with chronic periodontitis using area under curve analysis [31]. The optimal cut-off value of both miRNA 146a and miRNA 155 were found as reliable markers of periodontitis among diabetics and non-diabetics, which is in accordance with the findings of our study in non-diabetic group only as shown in S1 File. The differences in cut-off values and the sensitivity might be due to different sample type (GCF versus saliva) and different RNA extraction procedure.

MiRNA146a is encoded on human chromosome 5q33.3 [29] while miRNA146b is encoded by a separate gene on chromosome 10 which differs from miRNA-146a by 2 nucleotides at the 3’ end, and it is mostly dependent on IL-10 production after LPS challenge [34]. Xie et al demonstrated overexpression of miRNA 146 a/b in human gingival fibroblasts after stimulation with *P*. *gingivalis* LPS [35]. Overexpression of 146a/ b was associated with increased secretion of IL-1B, IL-6, and TNF-α in the LPS stimulated human gingival fibroblasts. Taganov et al found that miRNA 146a/b expression could be induced by many proinflammatory stimuli including TLR ligands in NF-kB-dependent manner [36]. Thus, miRNA146 may be involved in a negative feedback mechanism to regulate TLR signaling in response to bacterial products [37–39].

In the present investigation, it was found that both miRNA 146a & b expression were increased (12.3-fold and 4.3-fold respectively) among non-diabetic subjects with periodontitis when compared with those with healthy periodontium. The combined effect of diabetes and periodontitis is associated with a much higher increase in expression by 29.3 times. It is interesting to note that the effect of diabetes among those with periodontitis is not different from that of periodontitis among diabetics, which is a marginal increase in expression by 2.1 compared to 1.8. It is possible that diabetes and periodontitis share the same effect on this molecular pathway.

Interestingly, we found that the expression of miRNA146a was the highest among diabetic subjects with healthy periodontium compared to all the other study groups. This result was in accordance with that of Radovic et al who reported higher levels of 146a in GCF among subjects suffering from diabetes but having healthy gums compared to non-diabetics with healthy gums [31]. It has been suggested that overexpression of miRNA 146a in diabetic patients may represent a protective defense mechanism by the body against inflammation by downregulating TNF-α and other target genes of the NF-κB pathway; thus, protect the body against the complications caused by hyperglycemia [40]. Rong et al found upregulated plasma level of miRNA146a in diabetic patients and suggested that this expression could have predictive value for diabetes mellitus [41].

We also found that the expression of miR146a was reduced in diabetic patients with periodontitis compared to diabetic patients with healthy periodontium (Fig 3). This can highlight the clear fact that having periodontal disease may interfere with the body defense mechanisms in diabetic patients, making them more prone to further complications, such as ocular, cardiovascular and renal morbidities. It is well known that diabetes and periodontitis are linked by a two-way relationship. Diabetes increase the risk for periodontitis, and periodontal inflammation can negatively affect glycemic control [42].

**Fig 6:**
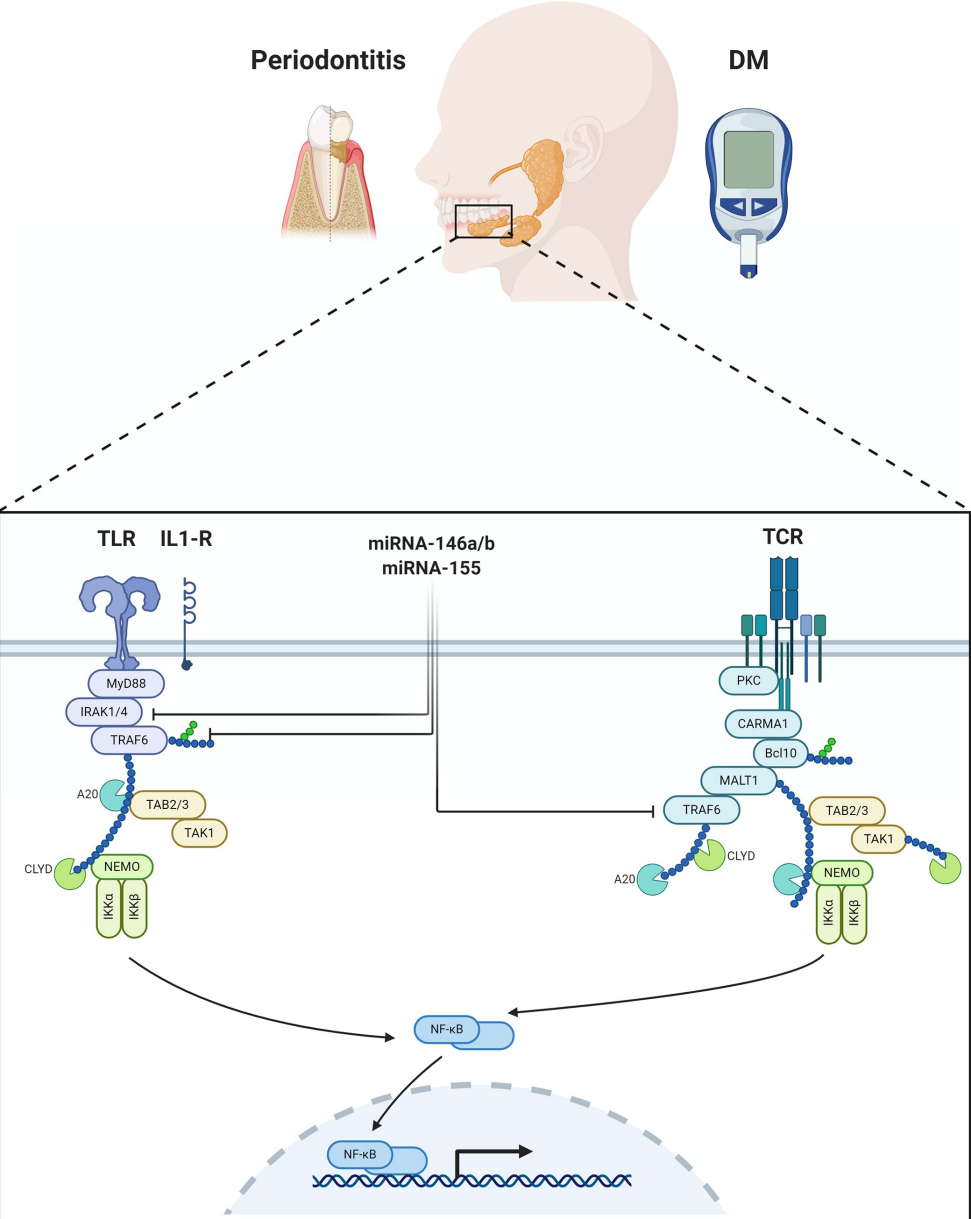
Mir 146a/b and Mir 155 regulatory network in diabetes mellitus and periodontitis.

MiRNA 155 is encoded by the host gene, within a region known as the B-cell integration cluster (BIC, miR155HG). Recently, it has been confirmed as a master regulator of inflammation, in the regulation of hematopoiesis, immune-regulation, in modulating humoral and innate cell-mediated immune responses. MiRNA 155 has also been demonstrated to inhibit Th2 differentiation by repressing IFN-γ. MiRNA155 was upregulated in the gingival tissues of periodontitis patients compared to healthy control [43]. Nayar et al, suggested that miRNA-155 expression level may be increased at the commencement of infection course and then diminish during subsequent infections [8]. Radovic et al also concluded that miRNA 155 increases the expression of pro-inflammatory factors such as NF-κB, TNF-α and interferon β, via TAB2, thereby acting in defense against pathogens, with negative feedback on the immune system, thus protecting the host from potentially damaging over reaction [31] as shown in Fig 6.

In the present study, miRNA 155 expression were elevated by 16.5 folds among diabetics with healthy periodontium compared to non-diabetics. Some studies have shown that miRNA 155 is involved in the regulation of blood glucose homeostasis and insulin sensitivity. Experimental studies in mice have proven that overexpression of miRNA 155 can improve glucose tolerance and enhance insulin sensitivity [44]. As the patients recruited in our study are with controlled diabetes, over expression of miRNA 155 may be one of the body’s defense mechanisms against the disease and may indicate good response to their antidiabetic therapy. Further studies are required to prove whether salivary miRNA155 can be used as a prognostic biomarker to predict the response to anti-diabetic therapy [28].

MiRNA 203 may also play fundamental role in inflammation and correlate with LPS release [45]. It is also involved in periodontal diseases through its effect on gingival keratinocytes. MiRNA 203 is said to regulate the cytokine signaling pathway as part of negative feedback loop, a property with direct relevance to the periodontal disease status [46]. The expression of miR-203 in saliva in the present study was elevated about 4.9 folds among people with diabetes with healthy gum when compared with non- diabetics. Moreover, miRNA 203 expression is significantly elevated by 5.9 folds in saliva of diabetics with periodontitis when compared with diabetics with healthy gum, owing this miRNA an additional property to be a specific marker that differentiates diabetics from non-diabetics and to differentiate between diabetics with healthy periodontium with those with periodontitis. Some authors suggested that *P*. *gingivalis* upregulate miRNA-203 through protein kinase C activation of AP-1. However, the exact mechanism by which *P*. *gingivalis* modulates miRNA-203 is as yet unknown [47]. The studied miRNAs in saliva could predict periodontal disease progression among diabetic patients but cannot predict diabetes from periodontitis as areas under curve were not significant for all miRNAs. Further studies are required to explore the effect of age as well as other clinical parameters on the chosen biomarkers on a larger cohort and to elucidate the mechanism of action of different miRNAs in relation to both periodontal inflammation and diabetes.

## Conclusion

The present study provided evidence that salivary levels of miRNA-146a/b, miRNA-155, and miRNA-203 could be considered as potential biomarkers for periodontal disease progression in nondiabetic individuals and could serve as potential biomarkers for diabetic periodontitis.

Understanding the miRNAs regulatory networks may provide additional opportunity for diagnosis and monitoring of the progression of many inflammatory-related diseases. The current study is exploratory by design. The biomarkers investigated were chosen as potentially useful, but the discriminatory nature of the potential biomarkers was not verified using an independent cohort of participants. The results from this study warrants the design of a follow up research study to verify these findings.

## Supporting information

S1 File (DOCX)
