# Supplementary material for: Salivary microRNA 155, 146a/b and 203: A pilot study for potentially non-invasive diagnostic biomarkers of periodontitis and diabetes mellitus
Source: PLoS One. 2020 Aug 5;15(8):e0237004. doi: 10.1371/journal.pone.0237004 (PMC7406085; doi:10.1371/journal.pone.0237004)
Supplement: S1 File — (DOCX) [file pone.0237004.s001.docx]

S1 Table. Bioinformatics tools used in the study

| Dataset | The Link |
| --- | --- |
| Human microRNA Disease Database | <http://www.cuilab.cn/hmdd> |
| FANTOM | <https://fantom.gsc.riken.jp/> |
| Enricher | <https://amp.pharm.mssm.edu/Enrichr/> |
| target scan tool | <http://www.targetscan.org/vert_72/> |
| DAVID Bioinformatics Resources 6.8, NIAID/NIH Functional Annotation Tool | <https://david.ncifcrf.gov/summary.jsp> |


**S2 Table. Comparison of accuracy of miRNA 146a and miRNA155 between Radovic et al study and the present study**

| Biomarker | AUC | | Cut-off | | Sensitivity | | 1-specificity | |
| --- | --- | --- | --- | --- | --- | --- | --- | --- |
|  | Present study | Radovic Study | Present study | Radovic Study | Present study | Radovic Study | Present study | Radovic Study |
| miRNA-146a (Periodontitis) | 0.72 | 0.99 | 11.04 | 4.11 | 0.84 | 1.00 | 0.12 | 0.04 |
| miRNA-146a  (Periodontitis with diabetes) | 0.82 | 0.95 | 6.21 | 6.21 | 0.71 | 0.88 | 0.4 | 0.04 |
| miRNA-155 (Periodontitis) | 0.86 | 0.93 | 8.97 | 8.97 | 0.87 | 0.96 | 0.21 | 0.21 |
| miRNA-155  (Periodontitis with diabetes) | 0.65 (NS) | 0.98 | 11.70 | 11.70 | 0.60 | 0.92 | 0.14 | 0.00 |
